# Long RP tachycardia with fusion QRS during ventricular overdrive pacing: Diagnostic challenge of a bystander nodoventricular pathway

**DOI:** 10.1016/j.hrcr.2025.10.040

**Published:** 2025-11-03

**Authors:** Hironori Nakamura, Hidehira Fukaya, Megumi Toraiwa, Sho Ogiso, Naruya Ishizue, Junya Ako

**Affiliations:** Department of Cardiovascular Medicine, Kitasato University School of Medicine, Sagamihara, Japan

**Keywords:** Atrioventricular nodal reentrant tachycardia, Nodoventricular pathway, Supraventricular tachycardia, Dual-chamber entrainment, Fusion QRS, Ventricular overdrive pacing

**Key Teaching Points**

• Ventricular overdrive pacing (VOP)-induced QRS fusion, traditionally considered specific to orthodromic reciprocating tachycardia (ORT), can also manifest in atrioventricular nodal reentrant tachycardia (AVNRT) with a bystander nodoventricular pathway. Relying solely on QRS fusion to diagnose ORT may lead to inappropriate ablation strategies.
• Although a significantly prolonged post-pacing interval minus tachycardia cycle length (PPI-TCL) after VOP (292 ms in this case) makes ORT less likely, it is insufficient to differentiate AVNRT from other rare tachycardias, such as decremental accessory pathway-mediated ORT. A combination of multiple electrophysiological maneuvers is essential for an accurate differential diagnosis.
• Dual-chamber entrainment is a valuable technique that refines the interpretation of conventional PPI-TCL. As demonstrated in this case, a markedly prolonged corrected post-pacing interval from the ventricle (PPIv)-TCL (244 ms and 316 ms) strongly supports the diagnosis of AVNRT, enabling an accurate diagnosis even in the presence of a bystander pathway.

## Introduction

Long RP supraventricular tachycardia poses diagnostic challenges. Fusion QRS complexes during ventricular overdrive pacing (VOP) have traditionally been interpreted as supportive of orthodromic reciprocating tachycardia (ORT), suggesting direct ventricular participation in the reentrant circuit.^1^ However, the presence of a concealed nodoventricular pathway (cNVP) may mimic this finding, complicating differentiation from atrioventricular nodal reentrant tachycardia (AVNRT). We describe a case of AVNRT with a bystander cNVP in which constant QRS fusion during VOP led to a diagnostic dilemma, ultimately resolved using dual-chamber entrainment (DCE).

## Clinical case

A 50-year-old woman with doxorubicin-induced cardiomyopathy (left ventricular ejection fraction 45%) was referred for evaluation of recurrent palpitations and exertional dyspnea. Her medications included amiodarone, eplerenone, empagliflozin, bisoprolol, azosemide, and vericiguat.

**Figure 1:**
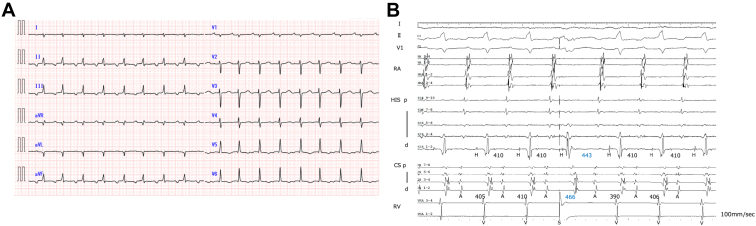
Intracardiac electrograms during tachycardia and the response to a His-bundle–refractory premature ventricular complex (PVC). **A,** 12-lead electrocardiogram during the electrophysiology study showing a narrow QRS tachycardia with a long RP interval (tachycardia cycle length 410 ms). **B,** Intracardiac electrograms demonstrating that the earliest atrial activation was recorded at the proximal coronary sinus. A His-bundle–refractory PVC delayed both A–A and H–H intervals without paradoxical reset.

During the electrophysiological study, a long RP narrow QRS tachycardia with a tachycardia cycle length (TCL) of 410 ms occurred spontaneously (Figure 1A). The earliest atrial activation was recorded at the proximal coronary sinus, and the atrial-His interval (AH) and His-atrial interval (HA) intervals were 181 and 222 ms, respectively. No AH block or His bundle dissociation was observed. A His-bundle–refractory premature ventricular complex (PVC) delayed both A–A and H–H intervals without paradoxical reset (Figure 1B).

**Figure 2:**
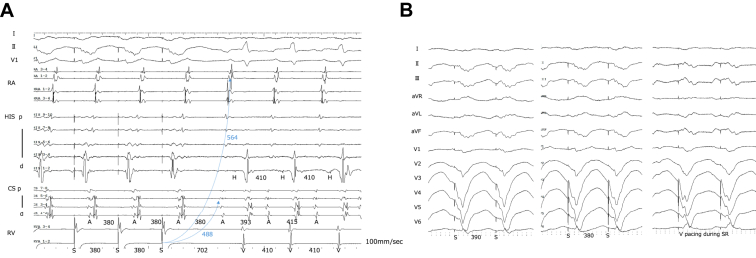
Ventricular overdrive pacing and fusion QRS. **A,** Ventricular overdrive pacing (VOP) from the right ventricular apex produced a V–A–V response with fusion QRS complexes, and the post-pacing interval minus tachycardia cycle length (PPI-TCL) was 292 ms. StimV→A intervals used for DCE were obtained during VOP: 564 ms from the high right atrium (HRA) and 488 ms from the coronary sinus (CS). These values, in combination with StimA→V and PPIa obtained from AOP (Figure 3), were used to calculate corrected post-pacing interval from the ventricle (PPIv)-TCL values. **B,** 12-lead electrocardiogram during VOP showing fusion QRS morphology. As the pacing rate was increased, the QRS complexes progressively approached the fully paced morphology.

**Figure 3:**
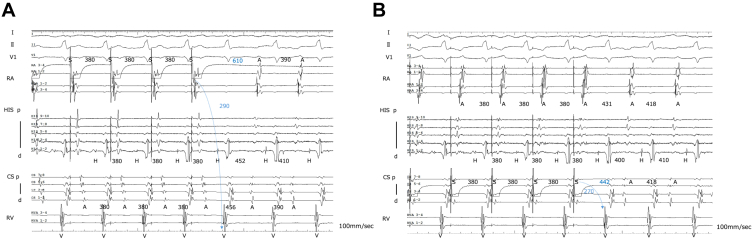
Atrial overdrive pacing (AOP). **A,** AOP from the high right atrium produced an A–H–A sequence without ventriculoatrial linking. The measured intervals were StimV→A 564 ms, StimA→V 290 ms, and post-pacing interval from the atrium (PPIA) 610 ms, yielding a calculated corrected post-pacing interval from the ventricle (PPIv)-TCL of 244 ms. **B,** AOP from the proximal coronary sinus also demonstrated an A–H–A response without VA linking. StimV→A was 488 ms, StimA→V 270 ms, and PPIA 442 ms, resulting in a corrected PPIv-TCL of 316 ms.

VOP from the right ventricular apex produced a V–A–V response with fusion QRS complexes, and the post-pacing interval (PPI) minus TCL was 292 ms (Figure 2A and 2B). Atrial overdrive pacing from both the high right atrium and the coronary sinus yielded an A–H–A sequence without ventriculoatrial (VA) linking (Figure 3A and 3B). Finally, DCE^2^ revealed markedly prolonged corrected post-pacing interval from the ventricle (PPIv)-TCL values: 244 ms from the high right atrium and 316 ms from the coronary sinus (Figures 2 and 3). Taken together, these findings favored AVNRT with a bystander cNVP rather than ORT. Based on these findings, slow pathway ablation was performed. The tachycardia was no longer inducible, and the patient has remained free of recurrence during follow-up.

## Discussion

AVNRT with a bystander cNVP is a rare arrhythmia that can closely mimic ORT, a diagnostic challenge particularly pronounced when, as in our case, VOP produces QRS fusion. Historically, ventricular fusion during entrainment was considered pathognomonic of ORT,^1^ a finding typically associated with a short PPI-TCL. Consequently, the combination of QRS fusion with a markedly prolonged PPI-TCL of 292 ms presented a significant diagnostic paradox that warrants detailed electrophysiological consideration.

**Figure 4:**
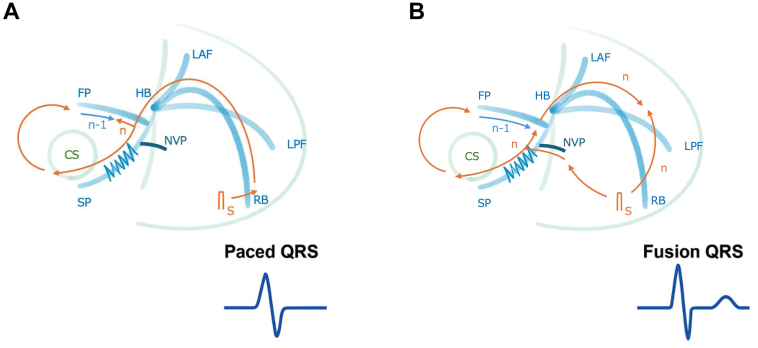
Schematic illustration of the proposed mechanism for QRS fusion during ventricular overdrive pacing in the presence of a bystander concealed nodoventricular pathway. **A,** When the pacing stimulus solely activates the ventricle via direct myocardial capture (or when the cNVP-HPS route is delayed), a fully paced QRS morphology results. **B,** Fusion QRS is produced by the collision of 2 distinct wavefronts originating from the same pacing stimulus (n). The first wavefront is from direct, slow myocardial activation from the pacing site. The second, concurrent wavefront is generated when the pacing stimulus propagates retrogradely through the bystander cNVP, accesses the His-Purkinje system (HPS), and then activates the ventricle rapidly and anterogradely. The parallel activation of the ventricle by these 2 wavefronts results in the fusion QRS morphology.

This apparent paradox can be resolved by a mechanism unique to this arrhythmia. The fusion likely results not from a collision between a preceding reentrant wavefront (n-1) and a pacing stimulus (n), but from the collision of 2 distinct ventricular wavefronts originating from the same pacing stimulus (n). The first wavefront is from direct myocardial capture at the pacing site, while the second propagates retrogradely through the bystander cNVP and then rapidly descends the His-Purkinje system (HPS) anterogradely (Figure 4). This mechanism requires 2 critical conditions: (1) the cNVP’s AV nodal insertion must allow for rapid electrical access to the HPS, and (2) antidromic capture of the His bundle from the pacing site must be sufficiently delayed—for example, by a functional retrograde block in the right bundle branch—to create the necessary time window for the cNVP-HPS wavefront to develop.

Crucially, the QRS morphology, determined by this parallel activation via the bystander cNVP, is dissociated from the PPI duration, which is independently determined by the slow conduction of the primary AVNRT circuit. This explains how QRS fusion and a long PPI-TCL can coexist, underscoring that fusion QRS during VOP is not specific for ORT when a bystander cNVP is present.

Although the hypothesis of AVNRT with a bystander cNVP provided a compelling explanation for the paradoxical findings, a definitive diagnosis required the rigorous exclusion of other tachycardias. The primary remaining concern was a rare but critical mimic known as decremental accessory pathway–mediated ORT (dAP-ORT).

A stepwise analysis of conventional pacing maneuvers effectively narrowed the differential diagnosis. The V–A–V response during VOP excluded atrial tachycardia, and the A–H–A sequence during atrial overdrive pacing excluded junctional tachycardia.^3^ Furthermore, the absence of VA linking made typical AV accessory pathway–mediated ORT less likely. This process left 2 main possibilities, which were dAP-ORT and AVNRT with a bystander cNVP. A His-bundle–refractory PVC that reset both atrial and His potentials without paradoxical reset confirmed the involvement of an accessory pathway but crucially could not distinguish between these 2 final diagnoses.

The diagnostic dilemma was amplified because dAP-ORT can itself present with a significantly prolonged PPI-TCL, and this case lacked other definitive findings, such as AH block or His bundle dissociation, that would unequivocally rule out ORT.^4^^,^^5^

To resolve this final diagnostic dilemma, we employed DCE, as described by Kaiser et al.^2^ DCE corrects for conduction delays and refines PPI-TCL interpretation, specifically by adjusting for all decremental conduction within the reentrant circuit. In our case, corrected PPIv-TCL values of 244 and 316 ms far exceeded the 80-ms cutoff separating septal ORT from AVNRT, providing decisive confirmatory evidence, and strongly favoring the latter. Although NV pathways were not part of the original DCE validation, the large margin above this cutoff, combined with the absence of VA linking, made AVNRT with a bystander cNVP the most consistent diagnosis.

Nonetheless, interpretation of DCE in the setting of NV pathways requires caution. Their decremental, oblique conduction can prolong StimV→A or StimA→V, leading to overestimation of PPIv-TCL. Rarely, triple AV nodal pathways can yield a false-negative for ORT by artificially lengthening the calculated value, while conversion to NV-ORT during pacing may prolong PPI from the atrium and result in a false-positive for ORT. In addition, the resolution of intracardiac recordings here did not allow unequivocal identification of orthodromic His capture.

We acknowledge the following limitations of this case study. First, as a single case report, the findings have limited generalizability. This is compounded by the fact that VOP was performed solely from the right ventricular apex, which precluded a comparative analysis with pacing from the right ventricular base that could have provided further diagnostic refinement. Second, the interpretation of QRS morphology on a surface electrocardiogram is challenging, as it is difficult to distinguish true electrophysiological fusion from other rate-dependent variations. This ambiguity means the presence of the cNVP was inferred from a collection of electrophysiological responses rather than being confirmed by direct mapping or histological evidence. Finally, our diagnostic conclusion relied heavily on the DCE maneuver. While the results were decisive in this case, the original DCE validation study did not include tachycardias involving NVPs. Therefore, the application of this criterion in the setting of a cNVP, while compelling, warrants further validation in future studies.

From a clinical standpoint, distinguishing AVNRT with a bystander cNVP from ORT is essential. Misinterpreting fusion QRS as diagnostic of ORT may prompt unnecessary ablation attempts targeting a presumed septal accessory pathway. In reality, the therapeutic target is slow pathway modification, which is effective and avoids unnecessary risk. This case highlights the importance of integrating multiple pacing maneuvers in a stepwise approach to prevent misdiagnosis and ensure appropriate treatment.

## Conclusion

This case highlights that fusion QRS during VOP is not pathognomonic of ORT when a bystander NVP is present. DCE, when interpreted alongside His-refractory PVCs and atrial pacing maneuvers, can provide decisive evidence favoring AVNRT by definitively excluding dAP-ORT. Recognition of this pitfall is crucial to avoid misdiagnosis and inappropriate ablation strategies.
